# The Preparation of Dentin Matrix-Polycaprolactone Drug Carrier Scaffold and Its Application in Promoting Bone Formation

**DOI:** 10.7759/cureus.94094

**Published:** 2025-10-08

**Authors:** Yiqun Dong, Nenghui Sun, Hongyang Zhu

**Affiliations:** 1 College of Rehabilitation Medicine, Qilu Medical University, Zibo, CHN; 2 Office of Scientific Research, Qilu Medical University, Zibo, CHN; 3 School of Stomatology, Qilu Medical University, Zibo, CHN

**Keywords:** dentin matrix, differentiation, osteoblasts, polycaprolactone, proliferation

## Abstract

Background and objective

The bioactivity of natural plant extracts, such as Chinese herbal medicines, is often limited by their poor water solubility. Therefore, selecting an appropriate drug carrier is crucial for exerting the pharmacological effects of these medicines. This study aimed to fabricate a dentin matrix-polycaprolactone (DM-PCL) drug carrier scaffold and evaluate its effects on the proliferation and differentiation of osteoblasts, thereby providing experimental support for subsequent drug loading applications.

Methodology

The DM-PCL carrier scaffold was constructed using electrospinning technology, and its surface morphology was observed by scanning electron microscopy (SEM). Osteoblasts were cultured in vitro, and the osteogenic effects of the DM-PCL scaffold were compared with those of a pure PCL scaffold through CCK-8 cell proliferation assay, live/dead cell staining, alkaline phosphatase (ALP) activity assay, cytoskeleton staining, and analysis of Runx2 mRNA expression levels.

Results

CCK-8 assay and live/dead staining showed that the DM-PCL drug carrier scaffold significantly promoted osteoblast proliferation (p<0.05). ALP activity detection indicated that the scaffold also enhanced early osteogenic differentiation (p>0.05). Phalloidin staining revealed a higher number of cells in the DM-PCL group, with adequate cell spreading and slightly clearer intracellular network structure and actin arrangement compared to the PCL group.

Conclusions

Based on our findings, the DM-PCL drug carrier scaffold can promote the adhesion, proliferation, and differentiation of osteoblasts.

## Introduction

Natural plant extracts, such as those derived from Chinese herbal medicine, possess significant pharmacological benefits and a favorable safety profile with minimal toxic side effects, leading to their broad application in clinical practice. However, the practical use of these natural compounds is often hindered by inherent limitations, including poor water solubility, low stability, limited bioavailability, and rapid metabolism and clearance in vivo, which collectively restrict their clinical potential [1]. Studies have indicated that the introduction of a physical barrier between a bone defect and the surrounding soft tissue during implantation can effectively prevent soft tissue infiltration and maintain adequate space for osteogenesis [2]. Moreover, if such a barrier is functionalized with osteoinductive drugs or bioactive materials, it can significantly enhance bone integration and regeneration [3].

The demineralized dentin matrix (DDM) has been shown to contain a variety of bioactive molecules that stimulate bone regeneration and promote the proliferation and differentiation of osteoblasts [4]. Additionally, DDM contains hydroxyapatite with a calcium-to-phosphorus ratio closely resembling that of natural bone tissue, further supporting its role as an ideal scaffold material [5,6]. Previous work by our team has focused on characterizing the physical properties of dentin matrices with varying particle sizes and demineralization degrees [7]. Nevertheless, the particulate nature of DDM makes it prone to dispersion and difficult to shape, limiting its handling and clinical applicability. Therefore, fabricating DDM into a structured drug-carrier system could not only improve its usability but also enhance its osteogenic performance. In this study, we aim to develop a composite scaffold by combining DDM with polycaprolactone (PCL), a biodegradable polymer known for its favorable mechanical properties, and to systematically evaluate its bone-promoting effects both in vitro and in vivo.

## Materials and methods

Experimental equipment

The following devices were used: Laser scanning confocal microscope (Leica, Germany); scanning electron microscope (Hitachi, Japan); centrifuge table, low speed (Medbase, China); CO_2_ incubator (Medbase, China); microplate reader (HUVLN, China), etc.

Preparation of carrier scaffolds

Preparation of Demineralized Dentin Matrix (DM) Materials

The pre-treated maxillary incisors of New Zealand rabbits were decalcified using a hydrochloric acid (HCl) solution (concentration: 1 mol/L) for 45 minutes. After drying, the teeth were pulverized using a grinder. The resulting dentin fragments were repeatedly sieved through standard test sieves of varying apertures for four to five cycles to obtain autologous dentin particles with a particle size of less than 100 μm. This experiment has been approved by the Ethics Committee of Qilu Medical University, with the approval number: YXLL2025D077.

Preparation of Electrospun Fibers

PCL was weighed and placed in a glass bottle. DM particles, N, N-dimethylformamide (DMF), and chloroform were then added dropwise sequentially into the bottle. The mixture was stirred using a heated magnetic stirrer until the PCL was completely dissolved, followed by continued stirring overnight to obtain the electrospinning solution. A 10 mL syringe was fixed to a micro-syringe pump and filled with the electrospinning solution. A no. 9 needle (inner diameter: 0.6 mm) was connected to the syringe via a polyvinyl chloride (PVC) tube. The tip of the needle was attached to the positive electrode of a high-voltage DC power supply, while the collector plate was connected to the negative electrode and grounded. The electrospinning process was carried out under the following parameters: voltage: (25.0 ± 0.5) kV, flow rate: 3 mL/h, collecting distance: 20 cm, at room temperature and appropriate humidity. After electrospinning, the aluminum foil collector was placed in a vacuum drying oven and dried for 24 hours. Using the same method, a pure PCL electrospun scaffold (without DM) was also prepared [6].

Scanning electron microscopy (SEM) observation of prepared scaffolds

The membrane was cut into 5 × 5 mm pieces, which were subsequently mounted onto metal stubs. The samples were sputter-coated with gold and imaged under a scanning electron microscope at an accelerating voltage of 20 kV and a magnification of 500x.

Subculture of osteoblasts

The cells were cultured in α-MEM medium supplemented with 10% fetal bovine serum (FBS) and 1% penicillin/streptomycin, and maintained in a humidified incubator at 37 °C with 5% CO₂. The medium was changed every two days. Cells were passaged three times, and experiments were conducted during the logarithmic growth phase.

Cell proliferation assay by CCK-8

MC3T3-E1 cells were seeded in 96-well plates at a density of 3×10⁴ cells/mL. The cells were divided into two groups: the DM-PCL group and the PCL-only group. The corresponding scaffolds were placed in each well, followed by the addition of 200 μL of cell suspension per well. Five replicate wells were set up for each group. After one, four, and seven days of culture, the original culture medium was discarded under light-protected conditions. Following the manufacturer's instructions, the CCK-8 mixture was added to each well. The absorbance (OD) value of each well was measured at a wavelength of 450 nm using a microplate reader.

Live/dead staining of cells on scaffolds

Cells were seeded onto the drug carrier scaffolds in a 24-well plate at a density of 3×10⁴ cells/mL. After three days of culture, the cells were stained using a live/dead cell staining kit according to the manufacturer's instructions. Following a 30-minute incubation in the cell culture incubator, the samples were observed under a fluorescence microscope.

Alkaline phosphatase (ALP) activity assay

Cells were seeded in 6-well plates at a density of 3×10⁴ cells/mL in 1 mL of cell suspension per well, with three replicate wells per group (grouping as previously described). After 24 hours of incubation in a constant temperature cell incubator, the medium was replaced with osteogenic induction medium containing calcification-inducing supplements. The cells were cultured for one, four, and seven days. Subsequently, the culture medium was removed, and cell lysis buffer was added to each well to completely cover the cells. After thorough lysis, the supernatant was collected by centrifugation. Following the kit instructions, the OD at a wavelength of 405 nm was measured using a microplate reader.

Cytoskeleton staining

Cells were seeded in laser confocal dishes at a density of 3×10⁴ cells/mL in 1 mL of cell suspension per dish, with three replicate dishes per group (grouping as previously described). After 24 hours of incubation, the original culture medium was discarded, and the cells were washed with PBS and fixed with paraformaldehyde. Then, 200 μL of rhodamine-phalloidin solution (100 nM) was added to cover the bottom of each confocal dish, followed by a 30-minute incubation in the dark. The samples were washed three times with PBS and counterstained with DAPI. Finally, the stained cells were observed under a laser scanning confocal microscope.

Statistical analysis

Statistical analysis was performed using SPSS Statistics software version 21.0 (IBM Corp., Armonk, NY). Measurement data were expressed as the mean ± standard deviation (x̄ ± s). Differences between groups were analyzed by one-way analysis of variance (ANOVA). A p-value of less than 0.05 was considered statistically significant.

## Results

Observation of scaffold surface morphology by scanning electron microscopy

**Figure 1:**
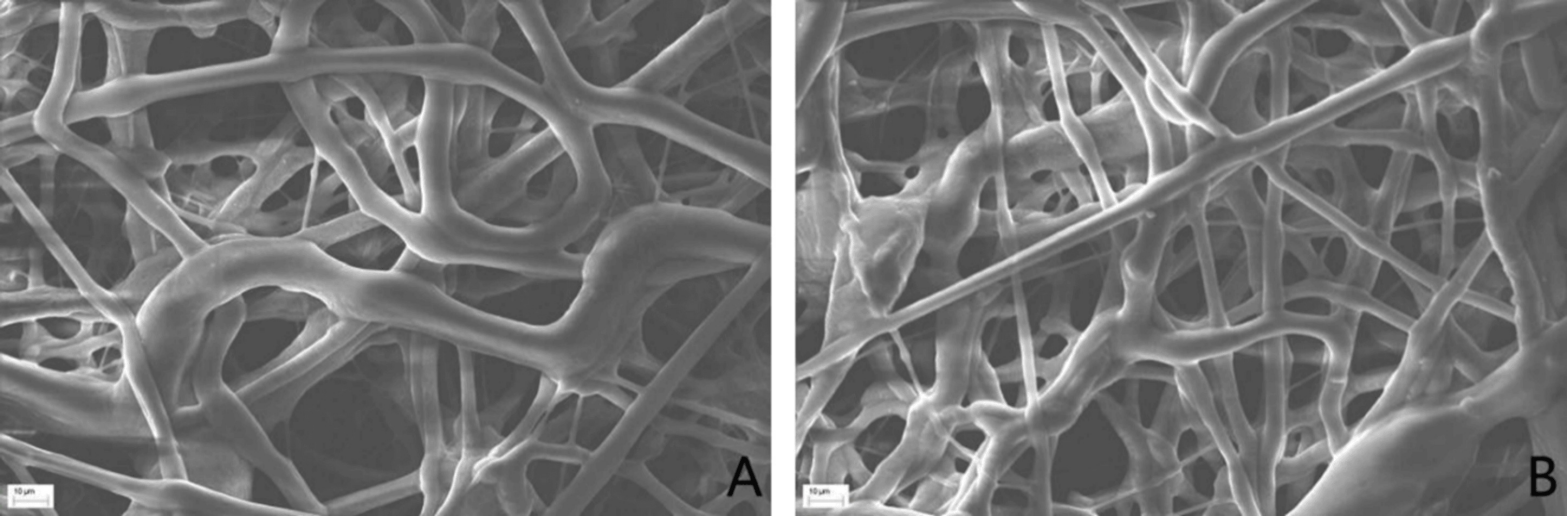
Scanning electron microscopy of drug carrier scaffolds A: PCL group. B: DM-PCL group PCL: polycaprolactone; DM: dentin matrix

Scanning electron microscopy revealed that the fibers of the PCL-only group exhibited a more uniform diameter and a relatively smooth surface. In contrast, the DM-PCL group showed greater heterogeneity in fiber diameter, with some fibers displaying particulate protrusions, confirming the successful incorporation of the DM into the PCL scaffold. Both types of scaffolds possessed a highly porous three-dimensional architecture, which is conducive to cell attachment and provides ample space for cell growth (Figure 1).

CCK-8 assay results

**Figure 2:**
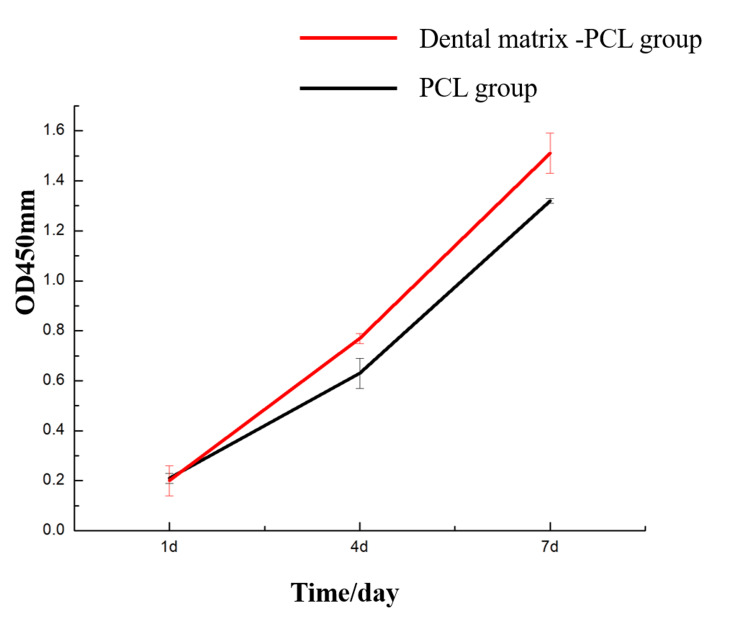
Osteoblast proliferation test results PCL: polycaprolactone

On day one of culture, no statistically significant difference in osteoblast proliferation was observed between the PCL-only group and the DM-PCL group (p>0.05).On days four and seven, the DM-PCL group showed a significant promotion in cell proliferation compared to the PCL group (p<0.05), as illustrated in Figure 2.

Live/dead staining of cells on scaffolds

**Figure 3:**
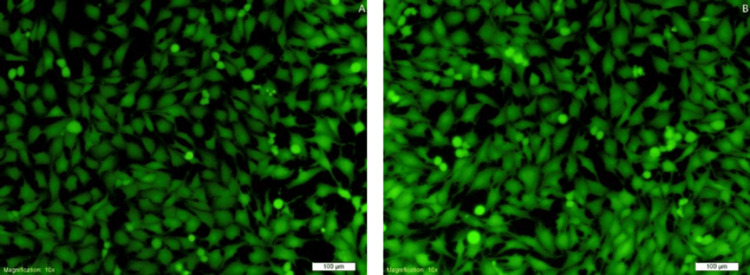
Cell live/dead staining on the scaffold A: PCL group. B: DM–PCL group PCL: polycaprolactone; DM: dentin matrix

In the images, green fluorescence indicates live cells, while red fluorescence indicates dead cells. Most cells in the polycaprolactone group and the DM-PCL group exhibited green fluorescence, with only a small number of dead cells observed. These results indicate that both types of drug-loaded scaffolds exhibit low cytotoxicity. The DM-PCL group showed stronger green fluorescence compared to the PCL group, suggesting that DM promotes cell proliferation. This finding is consistent with the results of the CCK-8 assay (Figure 3).

Results of alkaline phosphatase (ALP) activity assay

**Figure 4:**
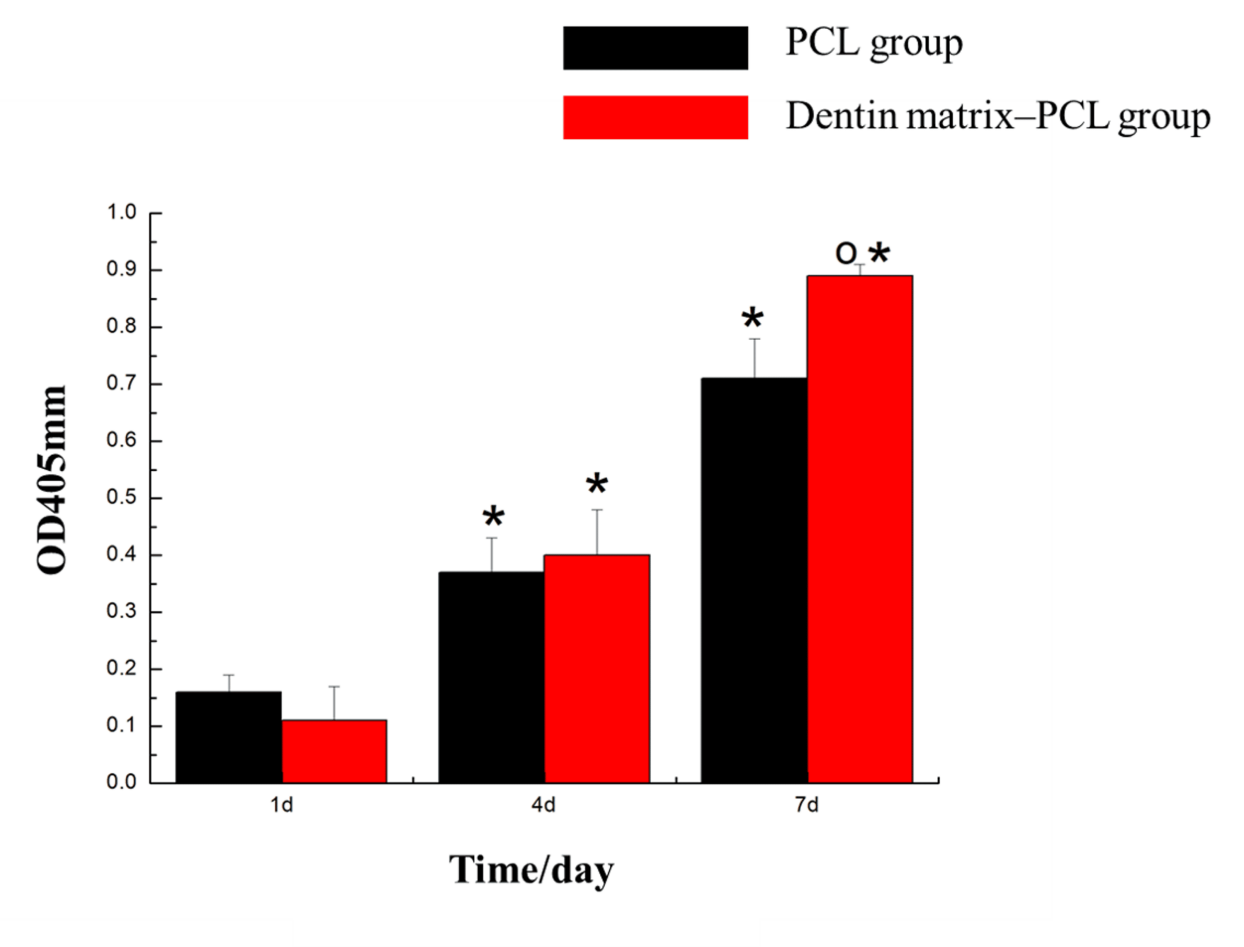
Alkaline phosphatase activity test results ^*^Compared with 1d, it has statistical significance (p<0.05. ^o^Compared with the blank control group, it has statistical significance (p<0.05) PCL: polycaprolactone

On day one and day four of culture, no significant differences in ALP activity were observed among the groups (p>0.05). On day seven, the DM-polycaprolactone group exhibited a significant increase in ALP activity compared to the PCL group (p<0.05), indicating that DM enhanced ALP activity (Figure 4).

Results of cytoskeletal staining

**Figure 5:**
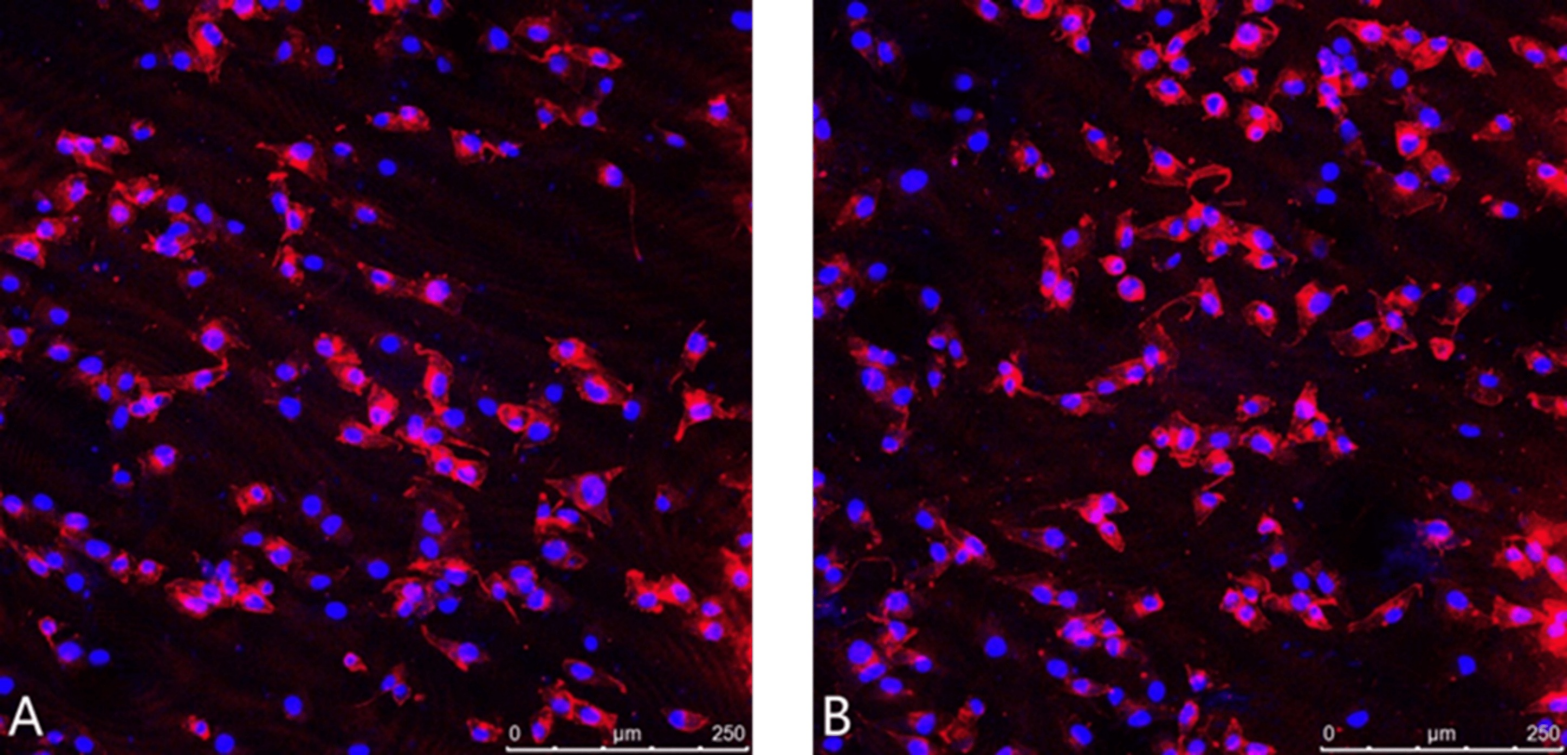
Cytoskeleton staining results A: PCL group. B: DM-PCL group PCL: polycaprolactone; DM: dentin matrix

Although the presence of the scaffold material resulted in a relatively high background signal, it was evident that the DM-PCL group exhibited a higher cell density and more cell spreading. The intracellular network structure and actin filament alignment appeared slightly clearer in the DM-PCL group compared to the PCL group (Figure 5).

## Discussion

In the field of bone regeneration, the application of traditional Chinese herbal medicine has become increasingly widespread. Maximizing the efficacy of these drugs is a major focus among researchers and experts. Studies have shown that the placement of a barrier membrane between the bone defect and soft tissue during implantation can isolate the soft tissue, thereby facilitating bone growth. Incorporating drugs or osteogenic substances into the barrier membrane can further accelerate the proliferation and differentiation of osteoblasts [2].

Studies have demonstrated that DDM contains bioactive substances capable of inducing bone regeneration and promoting the proliferation and differentiation of osteoblasts. DDM contains hydroxyapatite, which has a calcium and phosphorus composition similar to that of natural bone tissue, making it a suitable scaffold material [5]. Our research team has previously investigated the physicochemical properties of DDM with different particle sizes and degrees of demineralization [7]. In this study, DDM will be combined with PCL to fabricate a DDM-PCL composite scaffold. Its osteogenic effects will be evaluated to provide theoretical and experimental support for clinical applications.

SEM observations revealed that the PCL scaffold group exhibited more uniform fiber diameters and a relatively smooth surface. In contrast, the DM-PCL composite scaffold showed greater variation in fiber diameter, with granular protrusions present on certain fibers. These morphological features confirm the successful incorporation of DM into the PCL scaffold, providing a foundation for the subsequent release of bioactive factors from the DM to promote the proliferation and differentiation of osteoblasts. Both types of scaffolds possessed a loose and porous three-dimensional structure, which favored cell attachment and provided sufficient space for cell growth [8].

In the cell proliferation assay, after one day of culture, no statistically significant difference (p>0.05) was observed in the osteogenic cell proliferation capacity between the PCL group and the DM-PCL group. However, after four and seven days of culture, the DM-PCL group showed a significant increase in cell proliferation compared to the PCL group (p<0.05). These results indicate that the DM had no noticeable effect on promoting cell growth during the initial seeding stage. Over time, as bioactive substances were released from the DM, its osteopromotive effect increased, and the hydroxyapatite component also contributed to enhanced osteogenesis over prolonged culture.

In the live/dead staining assay, green fluorescence indicated live cells while red fluorescence represented dead cells. Both the PCL and DM-PCL groups showed predominantly green fluorescence with only occasional dead cells, demonstrating that both types of fabricated drug-carrier scaffolds possess low cytotoxicity. The DM-PCL group exhibited stronger green fluorescence, which is consistent with the CCK-8 assay results, collectively indicating that the DM promotes cell proliferation. The microsphere electrospun scaffolds prepared by Zhang et al. can also promote cell proliferation and differentiation [6].

The ALP activity assay showed no significant differences among the groups at one and four days of culture (p>0.05). However, after seven days of culture, the DM-PCL group exhibited significantly enhanced ALP activity compared to the PCL group. These results indicate that the DM did not markedly influence cell differentiation in the short term but promoted early osteogenic differentiation by day seven. This effect may be attributed to the level of early alkaline phosphatase expression [9]. The active substances in the DM that promote differentiation are closely related to hydroxyapatite [10]. The cytoskeleton plays a decisive role in the adhesion and spreading of osteoblasts [11,12]. Cytoskeletal staining revealed a higher cell count in the DM-PCL group, with adequate cell spreading and slightly clearer intracellular network structures and actin filament arrangement compared to the PCL-only group. This experiment demonstrates that DM promotes early cell adhesion and spreading.

In this experiment, a DM-PCL electrospinning scaffold was prepared and used for osteogenic research. We found that 3D porous carrier scaffolds can be prepared through electrospinning technology. The hydroxyapatite and other components of the DM itself can promote the proliferation and mineralization of osteoblasts, and promote early cell adhesion. This experiment also has certain limitations. For instance, the optimal particle size of DM and the best raw materials for electrospinning still need to be studied. Additionally, in the next step of the experiment, we will conduct in vivo experiments on animals to provide more data support for the application of drug carriers.

## Conclusions

Through cell proliferation and differentiation assays, this study confirmed that the DM-PCL carrier scaffold facilitates cell proliferation, differentiation, adhesion, and spreading. The innovative combination of DM and PCL forms a novel carrier material. Subsequent investigations by our research team will focus on the expression of osteogenesis-related genes and proteins, as well as characteristics such as drug loading capacity and sustained-release properties, to further refine the experimental data and provide theoretical support for clinical applications as a drug carrier scaffold.
